# Predictors of* Staphylococcus aureus* Colonization and Results after Decolonization

**DOI:** 10.1155/2016/4367156

**Published:** 2016-07-26

**Authors:** Tennison L. Malcolm, Le Don Robinson, Alison K. Klika, Deepak Ramanathan, Carlos A. Higuera, Trevor G. Murray

**Affiliations:** Department of Orthopaedic Surgery, Cleveland Clinic, 9500 Euclid Avenue, A41, Cleveland, OH 44195, USA

## Abstract

Protocols for the screening and decolonization of* Staphylococcus aureus* prior to total joint arthroplasty (TJA) have become widely adopted. The goals of this study were to determine: (1) whether implementation of a screening protocol followed by decolonization with mupirocin/vancomycin and chlorhexidine reduces the risk of revision compared with no screening protocol (i.e., chlorhexidine alone) and (2) whether clinical criteria could reliably predict colonization with MSSA and/or MRSA. Electronic medical records of primary patients undergoing TJA that were screened (*n* = 3,927) and were not screened (*n* = 1,751) for* Staphylococcus aureus* at least 4 days prior to surgery, respectively, were retrospectively reviewed. All patients received chlorhexidine body wipes preoperatively. Patients carrying MSSA and MRSA were treated preoperatively with mupirocin and vancomycin, respectively, along with the standard preoperative antibiotics and chlorhexidine body wipes. Screened patients were 50% less likely to require revision due to prosthetic joint infection compared to those not screened (*p* = 0.04). Multivariate regression models were poorly accurate in predicting colonization with MSSA (AUC = 0.58) and MRSA (AUC = 0.62). These results support the routine screening and decolonization of* S. aureus* prior to TJA.

## 1. Introduction

It has been reported that eight to nine percent of total joint arthroplasty (TJA) patients require revision within ten years of the primary surgery [1, 2]. Periprosthetic joint infection (PJI) following primary total hip arthroplasty (THA) and total knee arthroplasty (TKA) occurs in less than two percent of patients [3–5]. However, the rehabilitative course and complications associated with revision arthroplasty due to PJI are significantly more burdensome and the procedure is estimated to cost between three and four times more than primary TJA (i.e., ~$100000) [6–8].

*Staphylococcus aureus* and coagulase negative* Staphylococcus* are the leading causes of PJI in THA and TKA [9–11]. The anterior nares of 20–30% and two to six percent of American orthopedic patients are colonized by methicillin-sensitive (MSSA) [10–12] and methicillin-resistant* S. aureus* (MRSA) [11], respectively. The exact risk of PJI conferred by preoperative* S. aureus* colonization remains unclear. However, in orthopaedic surgery, preoperative MSSA colonization is associated with an added risk of surgical site infection, which is between two and nine times greater than among those patients who are not colonized [10]. It has been reported that colonization with MRSA affords a risk of surgical site infection up to four times higher than colonization with MSSA [13].

The exact mechanism by which nasal* S. aureus* colonization results in PJI is yet to be fully understood. In acute and early phases, it may result from the transient bacteremia observed after tracheal intubation [14]. Among patients who developed* S. aureus* surgical site infection, approximately 27–84% of identified causal organisms were identical to preoperatively isolated nasal strains [15, 16]. Alternatively, the anterior nares could serve as a reservoir for direct contamination of the surgical wound [17].

In recognition of the substantial morbidity associated with PJI, the high prevalence of* S. aureus* colonization, and a known association between preoperative* S. aureus* colonization and surgical site infection, nasal screening and decolonization have been widely adopted by the orthopedic community [10]. The routine use of preoperative chlorhexidine body wipes is intended to address this issue. In patients colonized with MSSA, topical mupirocin is applied twice daily for three to five days preoperatively. Additional measures (e.g., perioperative vancomycin) are taken for patients with MRSA. Combined decolonization of nasal and skin flora has been found to reduce the rate of surgical site infection by 77–200% [18, 19]. In a large (*n* = 42,531) multicenter prospective study, bundled preoperative nasal* Staphylococcus* decolonization, skin decolonization, and appropriate perioperative antibiotics were found to significantly decrease the rate of complex (deep incision or organ space) surgical site infection [20]. However, various studies have failed to show an added benefit of nasal decolonization alone [21, 22]. In addition, there is a paucity of evidence demonstrating that decolonization diminishes the risk for revision TJA.

The primary objective of this study was to test whether implementation of nasal screening and decolonization protocol prior to TJA results in reduced revision surgery secondary to PJI when compared with absence of such a screening protocol (i.e., routine use of chlorhexidine body wipes alone). A secondary objective was to determine whether clinical criteria could be used to reliably predict MSSA and/or MRSA colonization.

## 2. Methods

Beginning in 2011, as part of an initiative to improve patient outcomes, universal nasal screening for* S. aureus* and decolonization was implemented at select locations within the authors' hospital system. Universal screening was eventually expanded to the entire hospital system. However, the initial regional variability in protocol presented a unique opportunity to study the effects of universal screening by comparing two groups of patients who underwent TJA concurrently. This is a retrospective cohort study comparing screened patients and those not screened for* S. aureus* using nasal culture done at least four days prior to THA or TKA. As the concomitant use of nasal and skin decolonization was a part of quality measures to improve patient outcomes, no consent was obtained by patients to do so. Additionally, consent was not sought for inclusion in this particular study as it is retrospective and poses minimal harm; patient information is protected; and all data collected are to be in conglomerate deidentified manner. Those patients who cultured* S. aureus* were decolonized using topical mupirocin applied to the nares. Patients who underwent primary THA or TKA in any of the four major hospitals in our health system (Cleveland Clinic Foundation main campus, Hillcrest Hospital, Lutheran Hospital, and Euclid Hospital) between October 2011 and March 2014 were included in this study. Patients were considered “not screened” if they did not undergo nasal culture for* S. aureus* at least four days prior to TJA. Patients were excluded if they were found to be undergoing revision TJA and not primary TJA between 2011 and 2014. No other exclusion criteria were applied.

Patients were screened by sampling the nasal flora with a nasal swab and subsequent analysis with either PCR testing or bacterial cultures up to four weeks before surgery. Approximately one week prior to surgery, patients who carried* S. aureus* were treated with topical mupirocin twice daily for three days. All patients in the study used chlorhexidine body wipes preoperatively and received appropriate perioperative antibiotic prophylaxis. Patients not carrying MRSA received weight-based intravenous cefazolin 30 to 60 minutes preoperatively followed by repeated postoperative doses every eight hours for 24 hours. Patients who carried MRSA were administered weight-based vancomycin preoperatively followed by repeated postoperative doses every twelve hours for 24 hours. Those allergic to cephalosporin were administered either clindamycin or vancomycin in a similar manner.

The primary outcome was the incidence of revision arthroplasty following primary THA or TKA. Revision rates were assessed only in those patients with at least one year of follow-up (i.e., only surgeries performed between October 2011 and March 2013) and were stratified according to preoperative diagnosis. Demographic (i.e., gender and age) and clinical characteristics (i.e., body mass index, preoperative diagnosis, Charlson Comorbidity Index (CCI), and history of previous admissions) were also collected. For patients who underwent screening, MSSA and MRSA colonization was assessed. For patients who underwent revision for suspected infection, Musculoskeletal Infection Society (MSIS) criteria (e.g., evidence of a communicating sinus tract, isolation of pathogen from tissue/fluid cultures, inflammatory markers, synovial fluid analysis, presence of purulence in joint, and histologic analysis of periprosthetic tissue) were reviewed to confirm the diagnosis of PJI.

Predictors of* S. aureus* colonization were evaluated using the medical records of screened patients undergoing TJA who underwent surgery from October 2011 to March 2014. The associations of demographic, clinical variables, and screened status (screened and not screened) with* S. aureus* were assessed using Pearson's Chi-square test and Fisher's exact test for categorical variables and Student's* t*-test for continuous variables. Multiple logistic regression models were used to assess the combined effect of demographic and clinical variables on colonization rates. Variables considered for regression model entry are specified in the appendix. Individual comorbidities were individually represented in a bivariable fashion (i.e., 1/0). Gender, hospital site, previous inpatient stay within a specified period of time, and preoperative diagnosis were also represented in a bivariable manner. Charlson Comorbidity Index, age, and BMI were represented via continuous numerical variables. Because hospital site locations were mutually exclusive, only three variables were used to represent the four locations. It should be noted that there was a known discrepancy in staphylococcal screening across hospital sites over time. At the beginning of the study period, screening was universally encouraged at two of the four hospital locations. By the end of the study period, screening was universally encouraged in all hospital sites. This difference in practice over time in addition to the large cumulative rate of TJA across the four hospitals is what has enabled the present study to evaluate the impact on screening and postoperative outcomes. Time was not included in this model due to the limited timespan of the study (2.5 years). Regression models were constructed using backwards regression and the minimum Akaike information criterion (AIC) was the criterion for exiting the model. Regression model accuracy was judged using receiver operating characteristic (ROC) curves. As all available patients were included, calculation of the available power was based upon the size of the final cohort. All analyses were completed using JMP v7.0 (SAS Institute Inc., Cary, NC).

## 3. Results

Demographic and clinical characteristics of the study populations are summarized in Tables 1(a) and 1(b). A query of the electronic medical records identified 5,678 patients who underwent TJA from October 2011 to March 2014 and who met our study inclusion and exclusion criteria. A total of 3,927 patients (THA, *n* = 1,608; TKA, *n* = 2,319) underwent preoperative screening and, if indicated, decolonization prior to surgery (Table 1(b)). Of the 5,678 TJA patients, 4,042 (71.2%) had at least one year of follow-up and those who were screened (*n* = 2,291; THA *n* = 939; TKA *n* = 1,352) were compared with others who were not screened (*n* = 1,751; THA *n* = 700; TKA *n* = 1,051) (Table 1(a)). The remaining 1,636 (28.8%) were not lost to follow-up. Rather, at the time of study initiation, less than one year had passed since their index procedure and these patients were therefore excluded from analyses examining postarthroplasty failure.

Bivariable analyses showed that the two groups of patients were similar in most demographic (age and gender) and clinical (operative joint, BMI, and previous inpatient stay) characteristics. Patients who were not screened had, on average, higher CCI scores than screened patients (*t*-test, *p* < 0.01). Twenty percent of screened patients undergoing TJA were colonized with MSSA and five percent were colonized with MRSA. Carriers of* Staphylococcus aureus* were more likely to be male (*χ* ^2^, *p* < 0.01). Carriers of MRSA had, on average, higher CCI (*t*-test, *p* < 0.01) and BMI (*t*-test, *p* = 0.02) scores and were more likely to have had an inpatient stay between three months (*χ* ^2^, *p* = 0.01) and twelve months (*χ* ^2^, *p* < 0.01) prior to surgery compared to those who were not carriers.

As shown in Table 2, rates of revision arthroplasty were similar among patients who were screened and those who were not screened (*χ* ^2^, *p* = 0.17, power = 0.28). However, the risk of revision due to PJI was significantly higher in the group of patients who were not screened (*χ* ^2^, *p* = 0.04). Among patients who were screened and decolonized preoperatively, none who were colonized with MRSA required revision arthroplasty within the follow-up period. After screening and decolonization, there were no differences in overall revision rate (*χ* ^2^, *p* = 0.56), revision due to PJI (*χ* ^2^, *p* > 0.99), or revision due to mechanical failure (*χ* ^2^, *p* = 0.43) between patients colonized preoperatively with* S. aureus* and not colonized with* S. aureus*. Coagulase negative* Staphylococcus* was the most common infecting bacteria (*n* = 6), followed by* S. aureus* (*n* = 4) and enterococci (*n* = 3) (Table 3).

Demographic and clinical variables tested for association with* S. aureus* colonization by logistic regression modeling are described in the appendix. Decreasing mean age, male gender, and specific hospital locations increased the odds of MSSA colonization (log-ratio, *p* < 0.01) (Table 4). Congestive heart failure and previous inpatient admission within the past 12 months were strongly associated with MRSA colonization (*p* < 0.01). Chronic kidney disease was also significantly associated with MRSA colonization (*p* = 0.029). As a whole, multivariable regression models were poorly predictive of MSSA and MRSA colonization, with AUC = 0.58 and AUC = 0.62, respectively.

## 4. Discussion

Among patients undergoing TJA, the rate of nasal colonization with* S. aureus* has been reported between 20 and 30% [22].* Staphylococcus aureus* colonization is an important risk factor for PJI, as underscored by reported rates of surgical site infection up to nine times greater among carriers of* S. aureus* compared to noncarriers [10]. Assuming that the risks associated with* S. aureus* can be mitigated through prophylactic decolonization, Courville et al. [23] demonstrated the economic advantages of screening and/or decolonization in all patients undergoing TJA. However, the optimal method of minimizing the risk of PJI in this population has not been definitively determined.

The present study demonstrates a statistically significant decrease in revision arthroplasty due to PJI among patients undergoing TJA who were screened, decolonized, and treated with antibiotics for* S. aureus* colonization versus those who were not subjected to the nasal screening and decolonization process. Furthermore, this benefit is apparent even though chlorhexidine body wipes were used for all patients undergoing TJA in this study, regardless of screening status. This is an important finding as the use of preoperative chlorhexidine body wipes alone (prior to hospitalization) has been reported to significantly decrease surgical site infection by 66% to 100% [24, 25]. Other studies have shown improved surgical site infection rates among patients undergoing TJA who underwent nasal decolonization in concert with chlorhexidine body wipes. However, most studies have failed to show a benefit in decreasing PJI or a specific advantage in nasal and skin decolonization over skin decolonization alone [21, 22].

Due to the rarity of revision arthroplasty due to PJI, a very large sample size is required to demonstrate the utility of screening and decolonization. In a double-blinded, randomized controlled trial evaluating the effect of mupirocin alone in preventing surgical site infection (both superficial and deep), Kalmeijer et al. [21] found that mupirocin afforded a 0.9% difference in surgical site infection rate between the interventional and control groups, although the results did not achieve statistical significance. In a prospective cohort study of 7,019 elective orthopaedic surgeries (including arthroplasty, spine, and sports medicine procedures), Kim et al. [13] found a 57% relative decrease in the incidence of surgical site infections after implementation of a screening and mupirocin decolonization protocol (*p* < 0.01). However, the control population of that study did not receive chlorhexidine body wash. Similarly, Schweizer et al. [20] found a 52% reduction in the rate of deep tissue and organ space surgical site infection before and after the implementation of bundled nasal and skin decolonization via mupirocin and chlorhexidine body wipes, respectively.

Critics of screening and nasal decolonization have noted its added burden, citing the additional personnel required to collect and process nasal flora samples, the time required for follow-up on screening results, and the potential for the development of resistance to mupirocin [22, 26]. The results from this study indicate that patients'* S. aureus* status cannot be predicted based solely on patient characteristics, thereby implying necessary individual evaluation of each patient. Overall, the average cost of screening and decolonization has been estimated to be as high as $100 per procedure [23]. However, the average direct costs of revision arthroplasty due to PJI approximate $100,000 [6]. However, on average, with a 0.52% absolute reduction in risk of TJA failure due to PJI among screened patients, TJA failure is estimated to prevent TJA failure in one of every 191 patients who undergo prophylactic screening and decolonization. This protocol could potentially correspond to estimated cost savings of at least $500 for each patient in whom it is utilized. An additional potential drawback of nasal decolonization stems from its limited long-term efficacy.

Berthelot et al. [15] found that the risk of* S. aureus* surgical site infection following surgery was 2.8 times higher among orthopaedic patients with preoperative* S. aureus* nasal colonization. Price et al. [11] reported the risk of surgical site infection to be 6.9 times higher among patients found upon screening to have MRSA. The results of this study show equivocal differences in revision rates due to PJI between carriers of MSSA and those who are not carriers of MSSA. No revision arthroplasty was performed in carriers of MRSA. This apparent reduction in risk is presumably due to* S. aureus* decolonization with mupirocin, vancomycin, or both. It is interesting to note that only those patients who were not screened for* S. aureus* developed PJI due to MRSA. However, the small number of these observed events precludes further interpretation. These results support decolonization in all patients who are colonized with* S. aureus* and undergoing TJA. Also, it was found that MSSA and MRSA varied significantly with regard to demographic and clinical characteristics. However,* S. aureus* also exhibited significant regional variations and its colonization could not be reliably predicted. Despite statistical significance for certain characteristics, wide confidence intervals for multiple variables within the models indicate the difficulties in reliably predicting colonization. These findings suggest that all patients undergoing TJA should undergo preoperative* S. aureus* screening.

Colonization of the nares by* S. aureus* has previously described as persistent and intermittent [27]. As much as 60% of* S. aureus* carriers have been reported to be intermittent carriers [27]. Intermittent colonization has previously been shown to afford a risk of surgical site infection similar to noncolonization [28]. Previous studies suggest laboratory (e.g., number of confirmed nasal* S. aureus* colonization results, bacterial load) and clinical (e.g., obesity, insulin-dependent diabetes) data can be used to differentiate persistent carriers from intermittent carriers [29]. These are findings commensurate with those of this study as there was a significant association with BMI and increasing comorbid disease burden with* S. aureus* colonization. Due to the retrospective nature of this study and lack of longitudinal colonization data, a determination of persistent versus intermittent colonization cannot be made using this data. However, the role of selective decolonization of patients persistently colonized with* S. aureus* remains a future area of study.

Due to the retrospective, nonrandomized nature of this study, the findings are vulnerable to unmeasured bias. This study had a historical control group harbors' limitations associated with historical controls. Patients who were offered screening were more likely to have undergone surgery at a satellite hospital where the patient population is generally healthier with fewer medical comorbidities. However, the demographic and clinical similarities between patients who were screened and those who were not screened are suggested. Additionally, surgeons within the hospital system often operated at multiple hospital sites, thereby controlling for surgeon-dependent risk factors for failure and infection. Our follow-up interval was limited to one year in many patients and late infections (<2 years) may have been undetected [30]. However, late infections are generally not caused by* S. aureus* and thus not likely to be associated with* S. aureus* colonization [31]. Despite these limitations, the results add substantially to the growing body of evidence the fact that there is a significant benefit in decolonization of patients undergoing TJA with preoperative* S. aureus* colonization.

## 5. Conclusion

Colonization with MSSA or MRSA is an important predictor of surgical site infection and PJI in patients undergoing TJA. This study supports the use of a nasal swab screening protocol followed by the utilization of nasal mupirocin and targeted antibiotics in patients colonized with MSSA or MRSA. The findings demonstrate that* S. aureus* decolonization can be an effective strategy for reducing the revision rate due to PJI. A reliable predictive model using demographic and clinical characteristics could not be developed in this study. Due to the unpredictable nature of MSSA and MRSA colonization, it is reasonable to offer routine screening and decolonization to all patients undergoing TJA prior to surgery.

## Figures and Tables

**(a):** 

	Screened	Not screened	*p*
*n* (%)	2,291 (56.7%)	1,751 (43.4%)	
Mean age (SD)	63.8 (11.2)	64.2 (12.0)	0.24
Gender, *n* (%)			0.90
Female	1,352 (59%)	1051 (60%)	
Male	939 (41%)	700 (40%)	
Joint, *n* (%)			0.80
Hips	939 (41%)	700 (40%)	
Knees	1,352 (59%)	1,051 (60%)	
Mean Charlson Comorbidity Index (SD)	3.4 (1.7)	3.7 (1.9)	<0.01
Mean body mass index (SD)	32.1 (7.2)	31.9 (8.4)	0.43
Previous inpatient stay			
0-1 month, *n* (%)	115 (5%)	105 (6%)	0.17
1-2 months, *n* (%)	252 (11%)	201 (12%)	0.16
2-3 months, *n* (%)	367 (16%)	298 (17%)	0.48
3–6 months, *n* (%)	664 (29%)	508 (29%)	0.67
6–12 months, *n* (%)	1,054 (46%)	753 (43%)	0.03

**(b):** 

	MSSA	MRSA	Noncarrier	*p*
*n* (%)	798 (20.3%)	177 (4.5%)	2,952 (75.2%)	
Mean age (SD)	62.3 (11.4)	63.8 (11.5)	64.2 (10.9)	<0.01
Gender, *n* (%)				<0.01
Female	423 (53%)	99 (56%)	1,801 (61%)	
Male	375 (47%)	78 (44%)	1,151 (39%)	
Joint, *n* (%)				0.14
Hips	351 (44%)	76 (43%)	1,181 (40%)	
Knees	447 (56%)	101 (57%)	1,771 (60%)	
Mean Charlson Comorbidity Index (SD)	3.3 (1.7)	3.6 (1.8)	3.5 (1.6)	<0.01
Mean body mass index (SD)	32.4 (7.3)	32.9 (8.0)	31.8 (7.1)	0.02
Previous inpatient stay				
0-1 month, *n* (%)	32 (4%)	7 (4%)	148 (5%)	0.72
1-2 months, *n* (%)	72 (9%)	23 (13%)	295 (10%)	0.27
2-3 months, *n* (%)	112 (14%)	41 (23%)	413 (14%)	0.01
3–6 months, *n* (%)	200 (25%)	67 (38%)	768 (26%)	<0.01
6–12 months, *n* (%)	319 (40%)	97 (55%)	1,210 (41%)	<0.01

MSSA: methicillin-sensitive *Staphylococcus aureus*; MRSA: methicillin-resistant *Staphylococcus aureus*.

**Table 2:** Screening status of all total knee and total hip patients between October 2011 and March 2013 (*n* = 4,042) who subsequently underwent revision surgery (*N* = 47). A subgroup analysis of the screened group compared those colonized with MSSA (methicillin-sensitive *Staphylococcus aureus*) with those who were noncarriers. *p* values relevant to the subgroup analysis are denoted with an asterisk.

	Screened (*n* = 2,291)	Not screened (*n* = 1,751)	*p* value
Total revised (%)	22 (1.0%)	25 (1.4%)	0.17
*MSSA*	*4 (1.2%)*	—	*0.56* ^*∗*^
*Noncarrier*	*18 (1.0%)*	—	
Reason for revision			
Prosthetic joint infection (%)	9 (0.4%)	16 (0.9%)	0.04
*MSSA*	*1 (0.3%)*	—	
*Noncarrier*	*8 (0.4%)*	—	>*0.99* ^*∗*^
Mechanical failure (%)	13 (0.6%)	9 (0.5%)	1.0
*MSSA*	*3 (0.9%)*		
*Noncarrier*	*10 (0.5%)*		*0.43* ^*∗*^

**Table 3:** Frequencies of organisms isolated from culture specimens, grouped by screening status.

Bacterial Isolate^**∗**^	Screened	Not screened
MSSA	1	1
MRSA	0	2
CNS	4	2
*Enterococcus*	0	3
Other	3	5
No growth	2	4

MSSA: methicillin-sensitive *Staphylococcus aureus*; MRSA: methicillin-resistant *Staphylococcus aureus*; and CNS: coagulase negative *Staphylococcus*.

^*∗*^Cultures of some patients were positive for more than one bacterial species.

**Table 4:** Estimated odds ratios for patient acquiring *S. aureus* colonization, according to final multivariate logistic regression models.

Model, independent variables	Estimated odds ratio	95% confidence interval	*p* value
*Methicillin-sensitive S. aureus*			
Age, per unit increase	0.988	(0.981, 0.995)	0.001^*∗*^
BMI, per unit increase	1.01	(1.00, 1.02)	0.578
CHF (versus no CHF)	0.662	(0.376, 1.10)	0.112
DM without complication (versus DM with complication)	0.707	(0.425, 1.12)	0.145
Female (versus male)	0.716	(0.610, 0.840)	<0.001^*∗*^
Hospital site #1 (versus hospital site #3)	0.720	(0.603, 0.858)	<0.001^*∗*^
Hospital site #2 (versus hospital site #3)	0.670	(0.526, 0.847)	<0.001^*∗*^
*Methicillin-resistant S. aureus*			
Infection (versus no infection)	0.101	(0.00951, 2.20)	0.119
CHF (versus no CHF)	0.298	(0.168, 0.568)	<0.001^*∗*^
DM without complication (versus DM with complication)	0.583	(0.309, 1.22)	0.144
CKD (versus no CKD)	3.72	(1.12, 23.1)	0.0289^*∗*^
Hospital site #3 (versus hospital site #2)	0.653	(0.410, 1.02)	0.0593
Hospital site #1 (versus hospital site #2)	0.677	(0.428, 1.04)	0.0783
Previous Inpatient stay within 1 year (versus none)	0.619	(0.448, 0.858)	0.0032^*∗*^

^*∗*^Denotes statistical significance.

Age and BMI were analyzed as continuous variables. All other factors were analyzed as categorical variables. Only those variables which met minimum Akaike information criterion are listed in this table.

BMI: body mass index, CHF: congestive heart failure, CKD: chronic kidney disease, and DM: diabetes mellitus.

**Table 5:** Initial variables for multivariable *S. aureus* colonization prediction model.

Comorbidity	Patient characteristics	Preoperative diagnosis
Myocardial infarction	Age	Avascular necrosis
Congestive heart failure	Female	Infection
Peripheral vascular disease	BMI	Unspecified Inflammation
Cerebrovascular disease	Hospital site #1	Fracture
Dementia	Hospital site #2	Tumor
Chronic pulmonary disease	Hospital site #3	Osteoarthritis
Rheumatoid arthritis	Hospital site #4	
Peptic ulcer disease	Previous IP stay within 1 month	
Mild hepatic disease	Previous IP stay within 2 months	
DM without complication	Previous IP stay within 3 months	
DM with complication	Previous IP stay within 6 months	
Hemiplegia/paralysis	Previous IP stay within 1 year	
Chronic kidney disease		
Malignancy except skin cancer		
Moderate/severe hepatic disease		
AIDS/HIV		
Charlson Comorbidity Index		

AIDS: acquired immune deficiency syndrome, BMI: body mass index, DM: diabetes mellitus, HIV: human immunodeficiency virus, and IP: inpatient.

## Appendix

See Table 5.
